# Parathyroid hormone-related protein levels and treatment outcomes in hypercalcemia of malignancy: a retrospective cohort study

**DOI:** 10.1093/jbmrpl/ziae178

**Published:** 2025-01-15

**Authors:** Kazuhiko Kato, Akio Nakashima, Ai Kimura, Yukio Maruyama, Ichiro Ohkido, Yoichi Miyazaki, Takashi Yokoo

**Affiliations:** Department of Internal Medicine, Division of Nephrology and Hypertension, The Jikei University School of Medicine, Tokyo, 105-8461, Japan; Department of Internal Medicine, Division of Nephrology and Hypertension, The Jikei University School of Medicine, Tokyo, 105-8461, Japan; Department of Internal Medicine, Division of Nephrology and Hypertension, The Jikei University School of Medicine, Tokyo, 105-8461, Japan; Department of Internal Medicine, Division of Nephrology and Hypertension, The Jikei University School of Medicine, Tokyo, 105-8461, Japan; Department of Internal Medicine, Division of Nephrology and Hypertension, The Jikei University School of Medicine, Tokyo, 105-8461, Japan; Department of Internal Medicine, Division of Nephrology and Hypertension, The Jikei University School of Medicine, Tokyo, 105-8461, Japan; Department of Internal Medicine, Division of Nephrology and Hypertension, The Jikei University School of Medicine, Tokyo, 105-8461, Japan

**Keywords:** PTHrP, hypercalcemia of malignancy, calcium, cancer, bisphosphonate

## Abstract

The challenge of managing acute hypercalcemia in patients diagnosed with hypercalcemia of malignancy (HCM) merits further attention. Elevated levels of PTHrP may be a risk factor for treatment resistance in acute hypercalcemia; however, few studies have tested this hypothesis. This study aimed to investigate whether high PTHrP levels represent an independent risk factor that impedes the treatment of acute hypercalcemia. This retrospective cohort study recruited 159 patients aged 20-80 years with diagnosed malignancies who had been hospitalized for hypercalcemia with PTHrP levels above the reference value (1.1 pmol/L). The median (25%-75%) patient age was 69 (61-76) years, and the median PTHrP level was 6.3 (3.3-11.1) pmol/L. The corrected calcium levels decreased from 12.8 mg/dL (11.9-14.1 mg/dL) to 10.6 mg/dL (9.8-11.7 mg/dL) following treatments, such as bisphosphonates and saline solution. Multivariate linear regression analysis showed less significant decreases in the corrected calcium levels as natural logarithm-transformed PTHrP levels increased (β coefficient [95% CI]: 0.569 [0.225-0.914]; *p* = .001 for Model 3). Multivariate logistic regression analysis showed an association between high natural logarithm-transformed PTHrP levels and lack of treatment response, defined as a corrected calcium level of ≤10.4 mg/dL in the last blood test conducted within 2 wk of treatment initiation (odds ratio [95% CI]: 0.504 [0.312-0.814]; *p* = .005). Therefore, elevated PTHrP levels are a potential risk factor for treatment resistance in hypercalcemia in HCM patients, complicating management regardless of calcium levels.

## Graphical Abstract

**Graphical Abstract:**
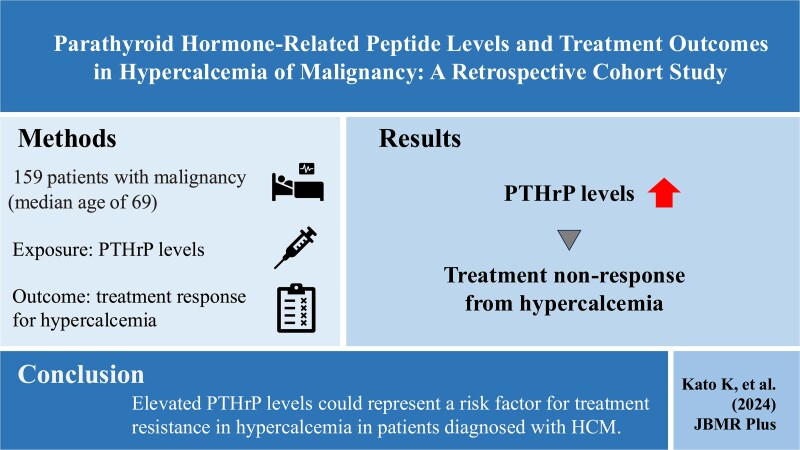

## Introduction

Hypercalcemia of malignancy (HCM) is a critical complication associated with malignancies such as renal, lung, and breast cancer, as well as multiple myeloma.^1^ It affects 20%-30% of patients diagnosed with advanced cancer,^2^ significantly influencing their prognoses.^3–9^ HCM is associated not only with long-term prognosis but also with oncological emergencies^10^^,^^11^ and a general decline in quality of life.^12^ The most common cause of HCM is PTHrP, which is responsible for 38%-69% of HCM cases, a much higher proportion than that associated with bone metastasis.^13^ PTHrP promotes bone resorption and hypercalcemia via RANKL-mediated osteoclast differentiation and activation.^14^ With the emergence of molecular targeted therapies and immunotherapies, there has been a paradigm shift in the prognosis of patients with cancer. The appropriate management of acute hypercalcemia represents a critical component of this new paradigm, as it helps position patients in optimal conditions to receive and derive maximum benefits from modern cancer treatment approaches.^15^^,^^16^

The challenge of managing acute hypercalcemia in patients diagnosed with HCM merits further investigation and currently suffers from an ambiguous characterization of the major risk factors that hinder its treatment. Insights from a large-scale 2015 observational study revealed that tumor site and the extent of disease progression represented the most critical determinants of therapeutic outcomes for HCM.^17^ Despite these advances, research concerning treatment-resistant hypercalcemia remains insufficient. A greater emphasis should be placed on the impact of clinical parameters, including PTHrP levels, on treatment outcomes. Theoretically, high PTHrP levels may represent a key independent risk factor hindering the treatment of acute hypercalcemia. Previous studies have suggested a relationship between PTHrP and resistance to treatment in hypercalcemia, although the data remain inconsistent,^18–22^ and studies testing this hypothesis remain lacking.

We have previously reported an association between PTHrP levels and long-term prognosis in patients with cancer.^9^ In this study, we retrospectively analyzed patients from this cohort who were treated for hypercalcemia during hospitalization and investigated the effect of PTHrP levels on hypercalcemia treatment.

## Materials and methods

### Study design

This retrospective cohort study aimed to clarify the association between PTHrP levels and hypercalcemia in patients with HCM. The primary exposure was the PTHrP level; the primary and secondary outcomes were the changes in corrected calcium (cCa) levels and treatment success for hypercalcemia, respectively. All procedures involving human participants were approved by the Institutional Review Board of The Jikei University School of Medicine (approval no.: 32-018(10093)) and were conducted in accordance with the 1964 Helsinki Declaration and its later amendments. The Institutional Review Board of The Jikei University School of Medicine waived the requirement to obtain informed consent from the patients, owing to the retrospective study design.

### Study population

Patients were recruited from the database of a previous retrospective cohort study,^9^ wherein the association between PTHrP and life outcomes was investigated between January 2000 and January 2020; patients who received treatment for hypercalcemia solely as outpatients were excluded. Therefore, this study included patients aged 20-80 yr with diagnosed malignancies who were hospitalized and treated for hypercalcemia with PTHrP levels above the reference value (1.1 pmol/L). We examined the relationship between PTHrP levels and treatment resistance in these patients over the 2-wk observation period following the initiation of treatment for hypercalcemia.

### Clinical and laboratory data collection

Demographic characteristics, comorbidities, and current medications were recorded at enrollment. Comorbidities and medications were determined via chart review during PTHrP measurement. Malignancy was defined based on a pathological diagnosis and did not include imaging examinations or clinical findings. PTHrP levels were measured using an immunoradiometric assay kit (SRL, Tokyo, Japan). The laboratory parameters that were measured included albumin (g/dL), creatinine (mg/dL), urea nitrogen (mg/dL), calcium (mg/dL), intact parathyroid hormone (pg/mL), and alkaline phosphatase (U/L). Alkaline phosphatase was measured using the method by the Japan Society of Clinical Chemistry. For Ca levels, the cCa level was calculated as follows if the serum albumin level was <4.0 mg/dL:^23^


$$ \mathrm{cCa}=\mathrm{Ca}+4.0\hbox{--} \mathrm{Alb} $$

Trends in cCa levels over 2 wk after treatment initiation were also investigated.

**Table 1:** Baseline patient characteristics (*n* = 159).

Variable		
**PTHrP, pmol/L**	6.3	(3.3-11.1)
**Age, yr**	69	(61-76)
**Sex, male, *n* (%)**	113	(71.1)
**BMI, kg/m^2^**	20.2	(17.5-23.2)
**Smoking history, *n* (%)**	83	(53.2)
**Cancer type**		
**Head and neck, *n* (%)**	28	(17.6)
**Lung, *n* (%)**	37	(23.7)
**Gastrointestinal, *n* (%)**	15	(9.4)
**Urinary tract, *n* (%)**	17	(10.7)
**Hematological, *n* (%)**	15	(9.4)
**Others, *n* (%)**	47	(30)
**Treatment of cancer**		
**Surgery, *n* (%)**	49	(32.2)
**Chemotherapy, *n* (%)**	105	(66.9)
**Comorbidities**		
**Diabetes mellitus, *n* (%)**	41	(46.6)
**Bone fracture, *n* (%)**	15	(17.1)
**Laboratory tests**		
**Albumin, g/dL**	2.7	±0.6
**Albumin <3 g/dL, *n* (%)**	108	(67.9)
**Creatinine, mg/dL**	1.0	(0.7-1.4)
**Creatinine ≥1.5 mg/dL, *n* (%)**	37	(23.3)
**Urea nitrogen, mg/dL**	21	(13-32)
**Calcium, mg/dL**	11.3	(10.5-12.9)
**Alkaline phosphatase, IU/mL**	343	(254-560)
**Intact parathyroid hormone, pg/mL**	8	(6-12)
**Treatment for hypercalcemia during the observation period**		
**Saline solution, *n* (%)**	111	(71.6)
**Diuretics, *n* (%)**	54	(34.4)
**Calcitonin, *n* (%)**	61	(38.9)
**Bisphosphonates (zoledronate), *n* (%)**	87	(55.1)
**Anti-RANKL monoclonal antibody, *n* (%)**	8	(5.4)
**Combined use of saline solution and bisphosphonates, *n* (%)**	66	(42.6)

Continuous variables are indicated as means (± SDs) and medians (interquartile ranges). Categorical variables are presented as numbers of patients (%).

### Statistical analyses

Data are presented as means ± SDs or medians with associated interquartile ranges. We demonstrated changes in cCa levels among the patients using box-beard diagrams (line: median, box: interquartile range). In addition, we illustrated the trends in cCa levels between 2 patient groups: one divided by the median PTHrP level (6.3 pmol/L) and the other with and without administering saline or bisphosphonate, the most selected treatments. We performed a linear regression analysis of natural logarithm-transformed PTHrP levels as continuous variables for changes in cCa levels between the baseline and the last blood sample within the observation period. For patients who died within 2 wk of the observation period, the last blood test before death was regarded as their last blood sample. The disjunctive cause criterion was used as the covariate selection method, and variables determined to be causes of exposures, outcomes, or both in previous studies were selected as confounders.^24^ The following models were created for the linear regression analysis: Model 1: sex, age, calcium levels, and albumin levels were added to the unadjusted model; Model 2: BMI and creatinine levels were added to Model 1; Model 3: cancer type and the administration of saline solution, bisphosphonates, diuretics, calcitonin, and anti-RANKL monoclonal antibodies were added to Model 2. We ensured that none of the variables exhibited multicollinearity. We also drew a restricted cubic spline curve showing the association between PTHrP levels and changes in cCa levels, adjusted using the explanatory variables of the multivariate linear regression analysis in Model 3. Three notches were used in the plot, and the dotted line indicates the 95% CI. To conduct a sensitivity analysis, multivariate logistic analysis was performed to examine the relationship between natural logarithm-transformed PTHrP levels as continuous variables and hypercalcemia treatment. We defined successful hypercalcemia treatment as a cCa level ≤ 10.4 mg/dL in the last blood sample tested within the observation period.^15^^,^^25^^,^^26^ Given the restriction on the number of variables that could be incorporated into the analysis, new models were constructed for the logistic regression analysis. Model 1 was created by adding sex, age, and calcium and albumin levels to the unadjusted model. In Model 2, we added cancer type and saline solution and bisphosphonates administration to Model 1. Finally, we conducted a subgroup analysis focusing on patients administered with saline solution and bisphosphonates combination treatment. In this group, we assessed the changes in cCa levels and performed the same multivariate linear regression analysis conducted in the primary analysis, limiting up to Model 2 due to the sample size. All tests performed in this study were 2-sided, with *p* < .05 indicating statistical significance. A complete case analysis was performed whenever any data were missing. As this study was exploratory in nature, no adjustment for multiplicity was planned. All statistical analyses were performed using Stata version 15.1 (StataCorp LLC).

## Results

### Patient characteristics

Among the 183 patients enrolled in the previous study,^9^ 24 treated solely as outpatients were excluded; we ultimately included 159 patients receiving hospital treatment for hypercalcemia. Table 1 lists their baseline characteristics. The median (25%-75%) patient age was 69 (range, 61-76) years, and the median PTHrP level was 6.3 (3.3-11.1) pmol/L. The median BMI was 20.2 (17.5-23.2) kg/m^2^. The mean albumin, median creatinine, and median calcium levels were 2.7 (SD, 0.6) g/dL, 1.0 (0.7-1.4) mg/dL, and 11.3 (10.5-12.9) mg/dL, respectively. The primary treatments for hypercalcemia included saline solution (71.6%), diuretics (34.4%), calcitonin (38.9%), bisphosphonates (55.1%), and combination treatment of saline solution and bisphosphonates (42.6%).

### Calcium changes

**Figure 1:**
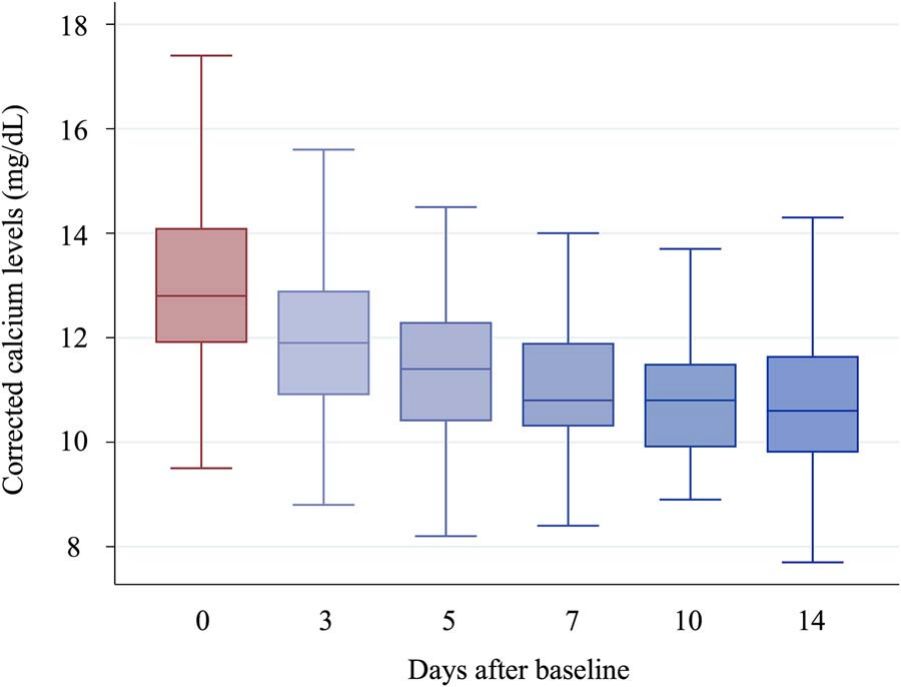
Changes in corrected calcium levels; line: median, box: interquartile range.

The patient cCa levels decreased from 12.8 (11.9-14.1) mg/dL to 10.6 (9.8-11.7) mg/dL. In the last blood test conducted during the observation period, 77 patients (48.4%) had recovered from hypercalcemia (Figure 1). As shown in Figure S1, the cCa levels decreased from 12.3 (11.4-13.1) to 10.3 (9.8-11.5) mg/dL in the lower PTHrP group (PTHrP level of ≥1.1 pmol/L but <6.3 pmol/L) and from 13.5 (12.3-14.9) to 11.1 (9.8-12) mg/dL in the higher PTHrP group (PTHrP level of ≥6.3 pmol/L) after 14 days of treatment for hypercalcemia. In the 2 groups divided by saline solution and bisphosphonate administration, we found a trend toward higher baseline cCa levels in the treatment group. However, these levels decreased to similar levels by Day 14 of treatment (Figures S2 and S3).

### Association of PTHrP levels with changes in calcium levels

**Figure 2:**
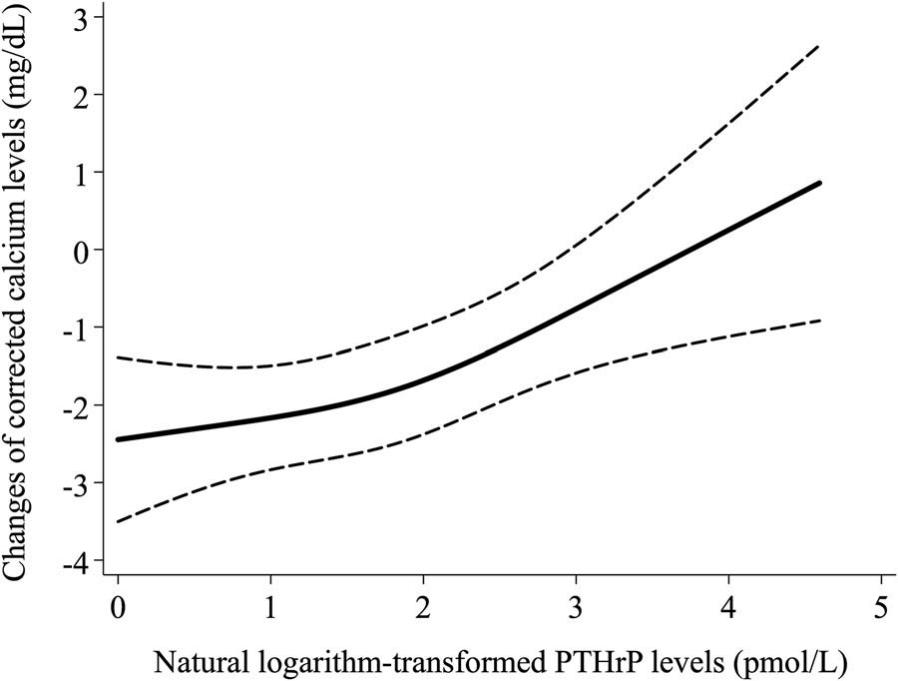
Restricted cubic spline curve showing the association between changes in corrected calcium levels and natural logarithm-transformed PTHrP levels. Three notches were used in the plot. The dotted line indicates the 95% CI.

Multivariate linear regression analysis of log(PTHrP levels) for changes in calcium levels (Table 2) showed that higher PTHrP levels were significantly associated with nonresponse to treatment (β coefficient [95% CI]: 0.569 [0.225-0.914]; *p* = .001 for Model 3). Figure 2 shows the restricted cubic spline curve adjusted using the variables in Model 3, indicating lower decreases in cCa as PTHrP levels increased.

**Table 2:** Univariate and multivariate linear regression analyses of log(PTHrP levels) for changes in calcium levels.

	*β* coefficient	(95% CI)	*p* value
**Unadjusted**	−0.23	(−0.634 to 0.156)	.234
**Model 1**	0.583	(0.281-0.884)	<.001
**Model 2**	0.58	(0.274-0.886)	<.001
**Model 3**	0.569	(0.225-0.914)	.001

PTHrP levels were log-transformed using the natural logarithm. Model 1: sex, age, calcium levels, and albumin levels were added to the unadjusted model. Model 2: BMI and creatinine levels were added to Model 1. Model 3: cancer type and the administration of saline solution, bisphosphonates, diuretics, calcitonin, and anti-RANKL ligand monoclonal antibodies were added to Model 2.

### Association of log(PTHrP levels) with improvement of hypercalcemia (sensitivity analysis)

Table 3 shows our univariate and multivariate logistic regression results with the improvement of hypercalcemia as the outcome. In our univariate analysis (odds ratio [95% CI]: 0.552 [0.379-0.806]; *p* = .002) and Model 2 (odds ratio [95% CI]: 0.504 [0.312-0.814]; *p* = .005), which included calcium levels and other potentially confounding factors, high PTHrP levels were found to be significantly associated with a lack of response to treatment.

**Table 3:** Univariate and multivariate logistic regression analysis of log(PTHrP levels) for improvement of hypercalcemia (decline in corrected calcium to ≤10.4 mg/dL).

	Odds ratio	(95% CI)	*p* value
**Unadjusted**	0.552	(0.379-0.806)	.002
**Model 1**	0.533	(0.35-0.812)	.003
**Model 2**	0.504	(0.312-0.814)	.005

PTHrP levels were log-transformed using the natural logarithm. Model 1: sex, age, calcium levels, and albumin levels were added to the unadjusted model. Model 2: cancer type and administration of saline solution and bisphosphonates were added to Model 1.

### Subgroup analysis of patients administered with saline solution and bisphosphonate combination treatment

The patient’s cCa levels decreased from 13.4 (12.5-14.8) to 10.6 (9.7-11.7) mg/dL following treatment (Figure S4). Multivariate linear regression analysis of log(PTHrP levels) for changes in calcium levels showed that, despite an associated trend, higher PTHrP levels did not significantly correlate with treatment nonresponse (Table 4).

**Table 4:** Univariate and multivariate linear regression analyses of log(PTHrP levels) for changes in calcium levels (patients administered with combination treatment of saline solution and bisphosphonates; *n* = 66).

	*β* coefficient	(95% CI)	*p* value
**Unadjusted**	−0.466	(−1.164 to 0.231)	.187
**Model 1**	0.37	(−0.124 to 0.865)	.139
**Model 2**	0.503	(−0.004 to 1.01)	.052

PTHrP levels were log-transformed using the natural logarithm. Model 1: sex, age, calcium levels, and albumin levels were added to the unadjusted model. Model 2: BMI and creatinine levels were added to Model 1.

## Discussion

This study included 159 patients with HCM and examined the hypothesis that PTHrP levels represent a risk factor for treatment resistance in cases of hypercalcemia. Our multivariate linear regression analysis showed that higher PTHrP levels were associated with a smaller decrease in cCa levels following treatment. In multivariate logistic regression analysis, higher PTHrP levels were found to be significantly associated with non-response to treatment. These findings suggest that PTHrP level likely represents a risk factor for treatment resistance in cases of hypercalcemia, independently of calcium level.

HCM is associated with hypercalcemia through various physiological mechanisms—notably, osteoclast activation caused by tumor-induced PTHrP secretion.^27^ PTHrP comprises 141 amino acids and acts by activating PKA and PKC by binding to PTH-PTHrP type 1 receptors.^28^ Although PTHrP is typically a locally produced growth factor, its dysregulated or unregulated systemic secretion by tumors increases osteoclastic bone resorption and calcium reabsorption in the renal tubules by binding to PTH-PTHrP type 1 receptors in the bones and kidneys.^29^ It has also been reported to induce hypercalcemia through a variety of mechanisms, including tumor cell proliferation, apoptosis, dormancy, and cachexia.^30–33^

HCM is typically treated using a combination of hypercalcemia-specific therapy and treatments for the underlying malignancy.^4^ The regimen often comprises saline solution, diuretics, bisphosphonates, and calcitonin. In this study, the most common treatments administered for hypercalcemia were saline solution in 111 (71.6%) patients and bisphosphonates in 87 (55.1%). When the trends in cCa levels were examined separately, with and without using these drugs, both showed a trend toward higher cCa levels at baseline in the treated groups that ultimately decreased to comparable levels after 14 d. However, clinicians are more likely to provide more robust treatments to patients with more severe cases of hypercalcemia, as a result of indication bias; therefore, the effects of these drugs could not be estimated from the results of this study.

Bisphosphonates represent the principal drugs used to treat HCM, given their effective suppression of osteoclast function and pronounced impact on severe hypercalcemia.^16^^,^^34^^,^^35^ Previous studies have suggested an association between PTHrP levels and resistance to bisphosphonate treatment. However, these studies did not consider calcium levels or include patients who were treated with zoledronic acid, which is the current standard for bisphosphonate therapy.^18–20^ In contrast, other studies have refuted any correlation between bisphosphonate resistance and PTHrP levels, indicating the need for further research regarding this relationship.^21^^,^^22^ This study contributes to this discussion by demonstrating a relationship between PTHrP levels and treatment resistance, independent of calcium levels, in a population where 50% received zoledronic acid.

Subgroup analysis of patients treated with the combination of bisphosphonate and saline solution, a highly effective therapy, demonstrated its sufficient reduction of calcium levels, even among those with elevated baseline cCa levels. Moreover, consistent with the findings in the overall cohort, higher PTHrP levels in this subgroup also tended to be associated with treatment resistance.

Recent studies have reported the potential efficacy of anti-RANKL monoclonal antibodies^36–39^ for treating hypercalcemia. Nevertheless, only 8 patients in this study (5.4% of the cohort) received anti-RANKL monoclonal antibodies.

A comprehensive observational study conducted in 2015 explored the relationship between various medications and hypercalcemia treatment outcomes.^17^ That study did not establish a significant correlation between the use of each specific drug and morbidity or mortality. Moreover, it lacked information regarding calcium levels, underscoring the need for subsequent research to determine the effectiveness of each drug in terms of managing acute hypercalcemia. This study aimed to assess the link between pharmaceutical interventions and successful treatment, yet faced challenges related to the inherent limitations of the study design, such as the observational and nonrandomized nature, as well as the potential for indication bias.

This study has limitations. First, although measuring cCa levels is a common clinical practice, it is often imprecise and can be misleading, particularly in patients with low albumin levels, making the measurement of bioactive ionized calcium preferable. Nonetheless, the assessment of ionized calcium was not feasible. Second, the retrospective nature of the study design precluded the examination of symptoms or electrocardiographic alterations attributable to hypercalcemia. Third, an inability to screen for alternative endocrine abnormalities or hormone-secreting neoplasms that present with hypercalcemia must be acknowledged despite the low prevalence of these conditions and their minimal anticipated impact on the analyses.^2^ Fourth, the heterogeneity of the cancer types among the study participants further complicates our understanding of hypercalcemia pathogenesis, which is likely to vary across different malignancies. Fifth, a lack of vitamin D level assessments represents another potentially significant omission. Sixth, we could not obtain data information regarding surgery and chemotherapy, which are fundamental treatments for malignancy; hence, these were excluded from the analysis. Furthermore, a lack of data on imaging impeded our ability to detect and assess bone metastases. Seventh, discrepancies may have existed between the timing of the initiation of hypercalcemia treatment and the onset of hypercalcemia. Additionally, this cohort included patients who died within the 14-d observation period. As outlined in the Methods section, the cCa levels from the last confirmed blood sample during the observation period were used for the outcome assessment of each case. Finally, only 71.6% of patients were administered saline solution, the primary treatment for hypercalcemia. It is possible that intravenous fluids with a lower sodium concentration were used or that priority was given to other medications, such as bisphosphonates. Additionally, this study may include some patients who were not administered saline solution due to reasons related to some pathological condition, such as a tendency to fluid overload. However, the specific reasons for the omission of saline solution in individual cases remain unclear due to the limitations of the research design.

This study proposes that PTHrP levels may represent a risk factor for treatment resistance in cases of hypercalcemia. Assessing PTHrP levels may help to predict treatment resistance when managing acute hypercalcemia in patients diagnosed with HCM. Therefore, patients with high PTHrP should be closely monitored for refractory hypercalcemia in approximately 14 d following the initial therapy.

## Supplementary Material

Supplemental_Figure_1_ziae178

Supplemental_Figure_2_ziae178

Supplemental_Figure_3_ziae178

Supplemental_Figure_4_ziae178

Supplemental_Caption_ziae178

## Data Availability

The datasets used in this study are available from the corresponding author upon reasonable request.
