# Supplementary figures and images for: Parathyroid hormone-related protein levels and treatment outcomes in hypercalcemia of malignancy: a retrospective cohort study
Source: JBMR Plus. 2025 Jan 15;9(3):ziae178. doi: 10.1093/jbmrpl/ziae178 (PMC11807284; doi:10.1093/jbmrpl/ziae178)

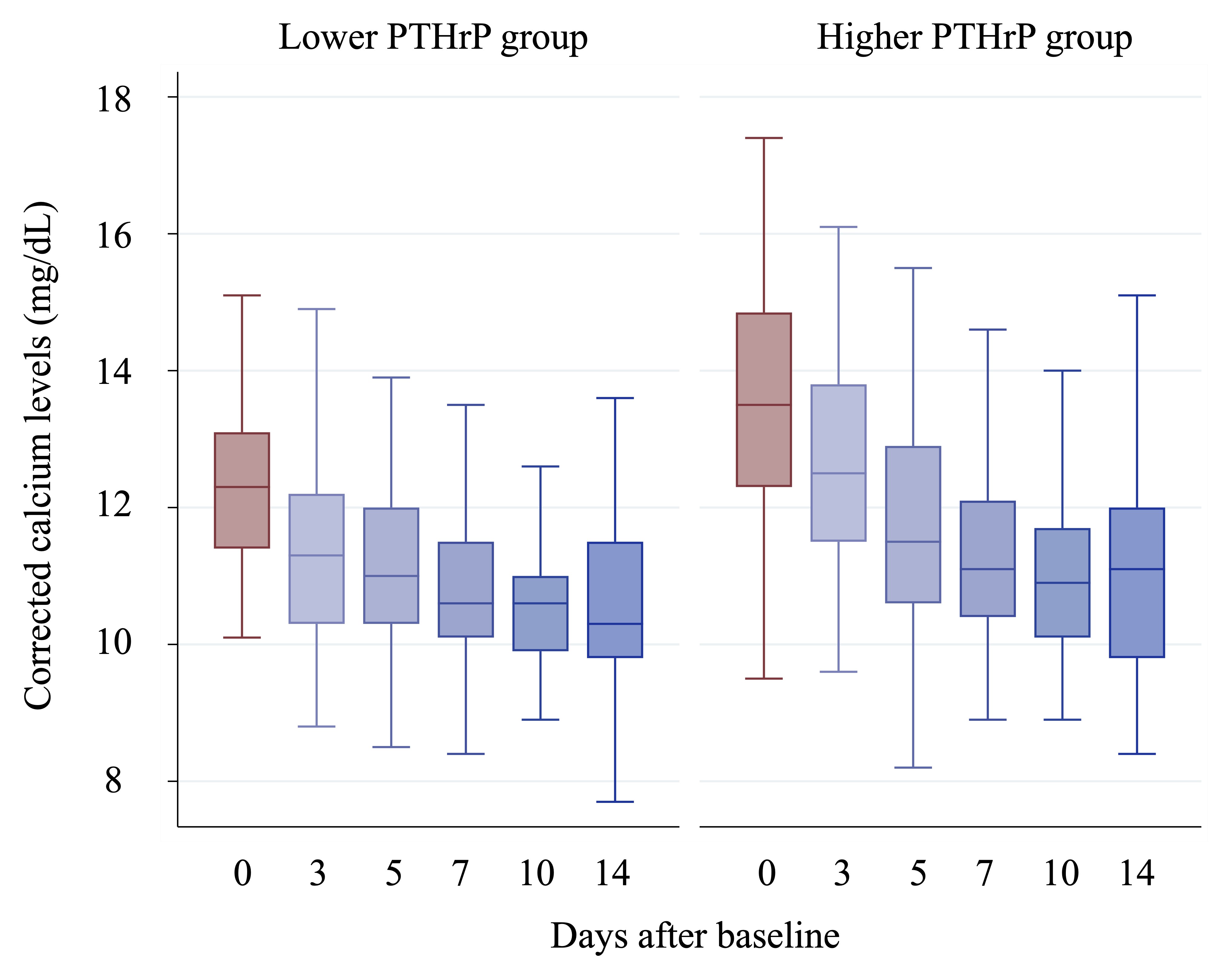

Supplement: Supplemental_Figure_1_ziae178 [file supplemental_figure_1_ziae178.jpeg]

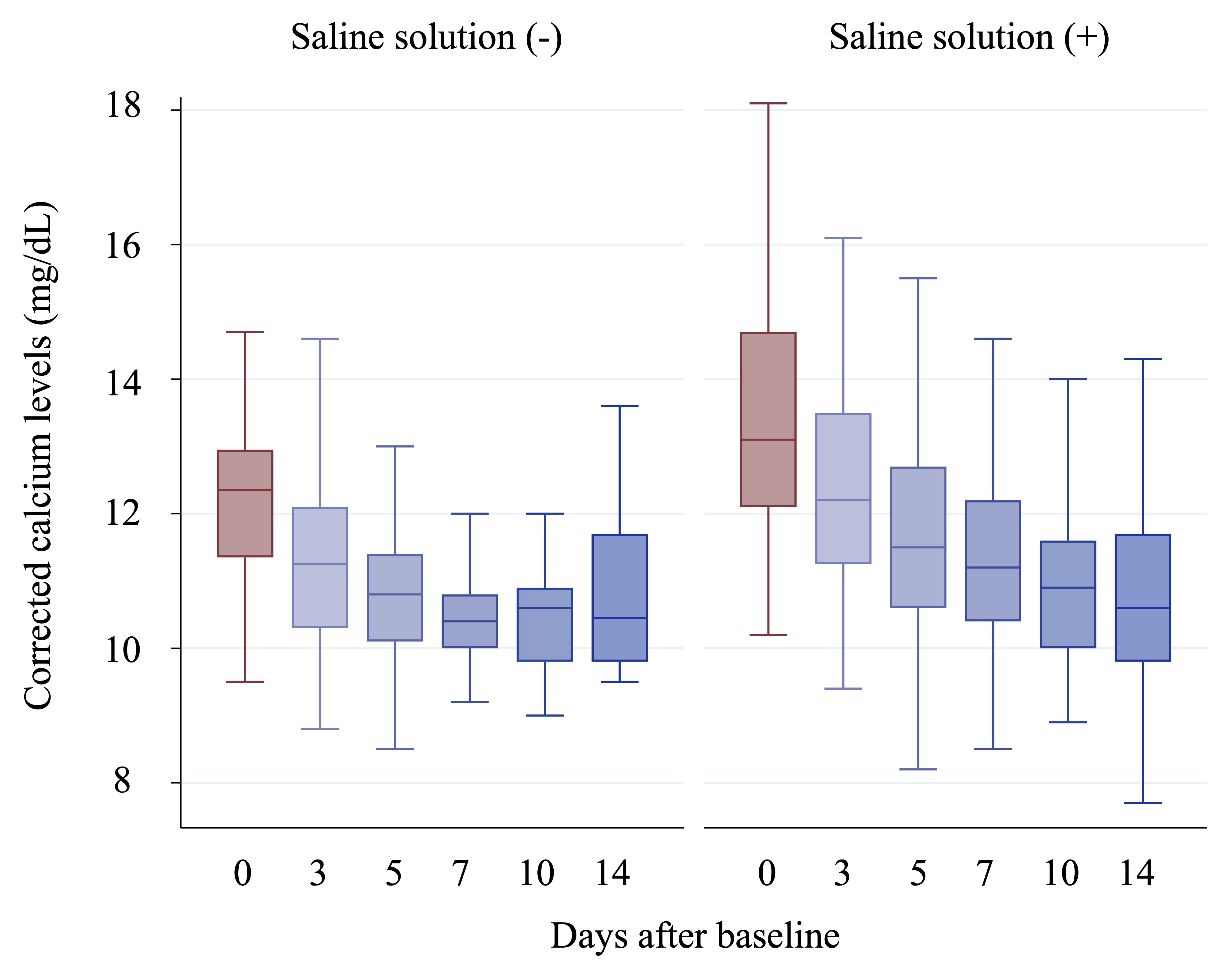

Supplement: Supplemental_Figure_2_ziae178 [file supplemental_figure_2_ziae178.jpeg]

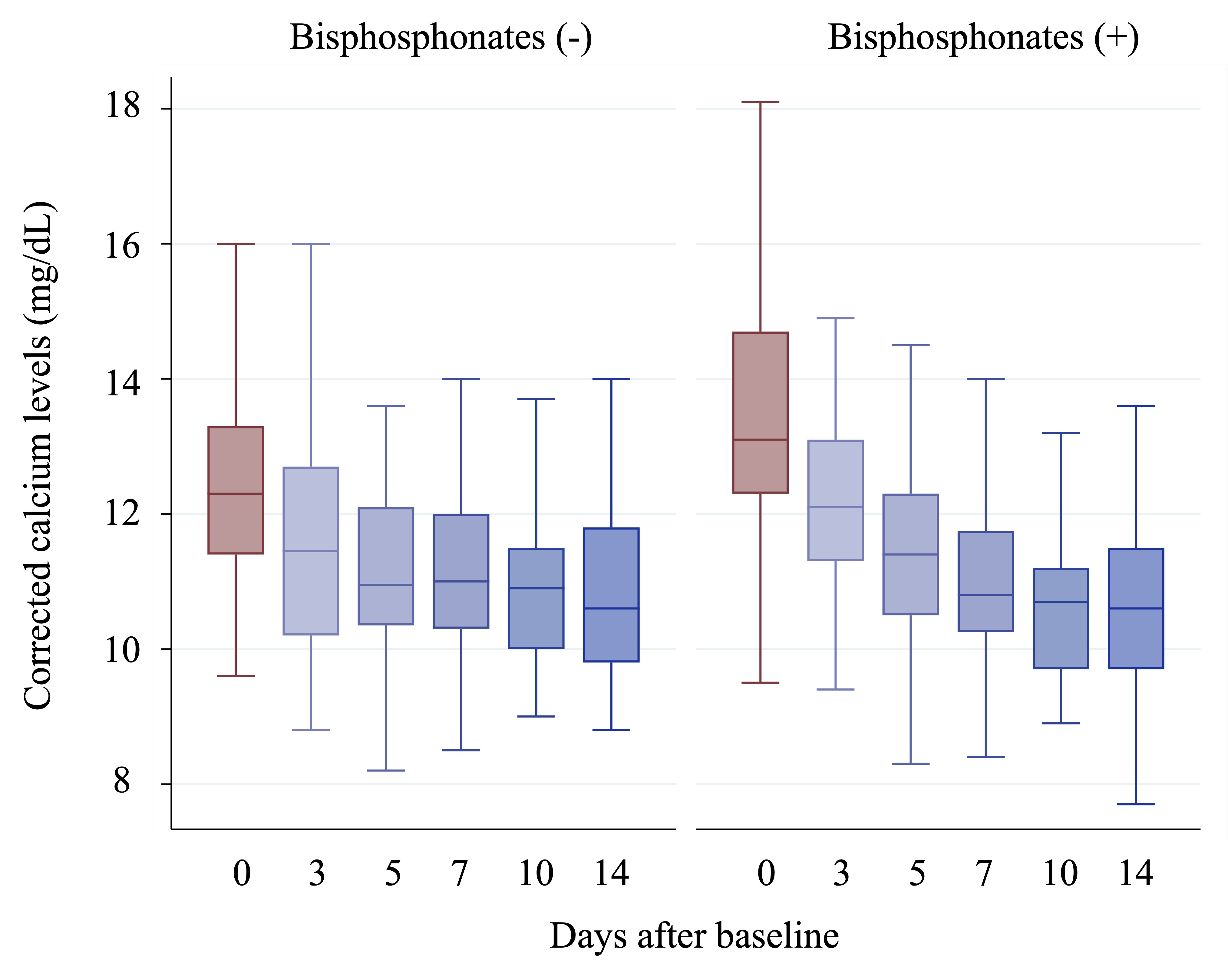

Supplement: Supplemental_Figure_3_ziae178 [file supplemental_figure_3_ziae178.jpeg]

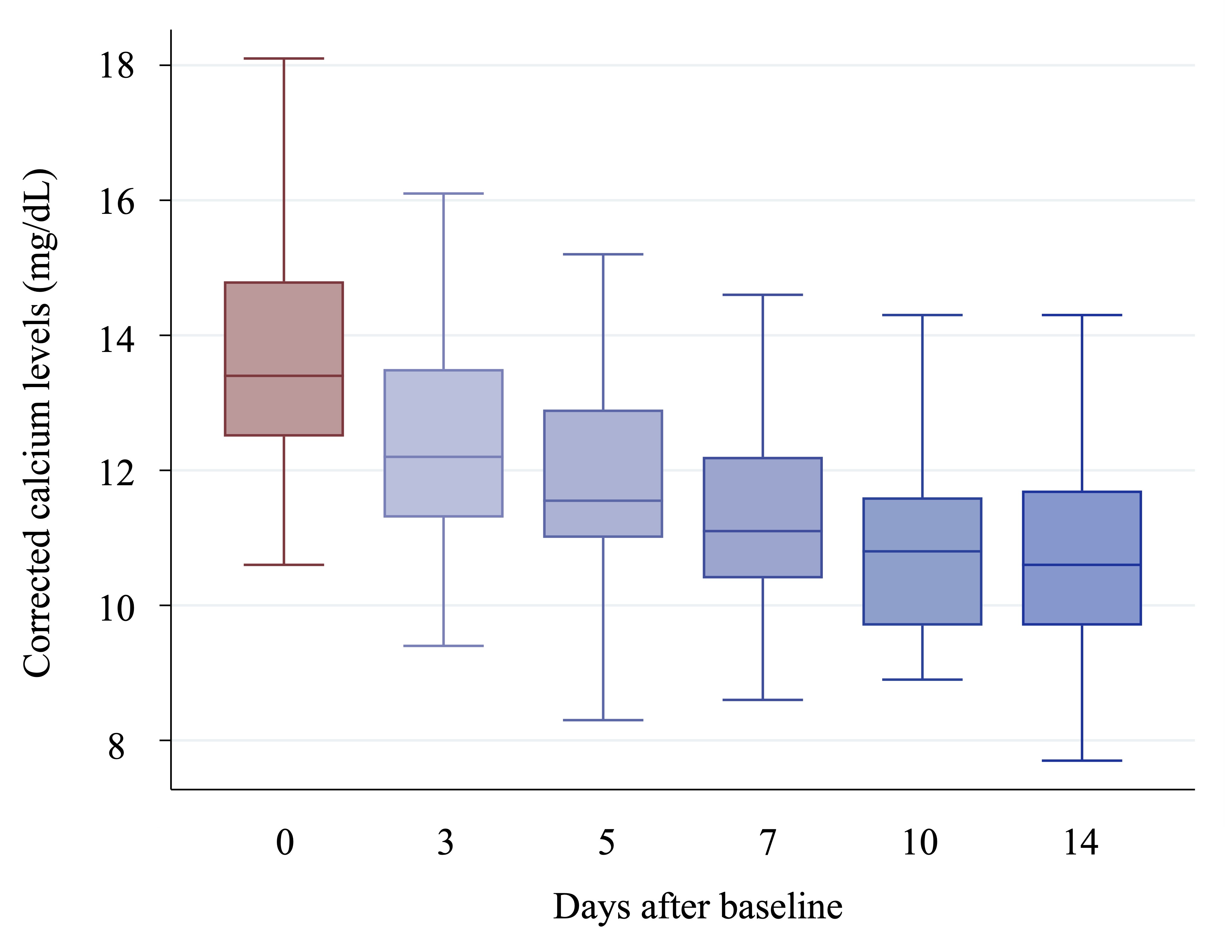

Supplement: Supplemental_Figure_4_ziae178 [file supplemental_figure_4_ziae178.jpeg]
