# Supplementary material for: Parathyroid hormone-related protein levels and treatment outcomes in hypercalcemia of malignancy: a retrospective cohort study
Source: JBMR Plus. 2025 Jan 15;9(3):ziae178. doi: 10.1093/jbmrpl/ziae178 (PMC11807284; doi:10.1093/jbmrpl/ziae178)
Supplement: Supplemental_Caption_ziae178 [file supplemental_caption_ziae178.docx]

Supplementary Figure 1. Changes in corrected calcium levels divided by parathyroid hormone-related protein levels. PTHrP, parathyroid hormone-related protein.

Supplementary Figure 2. Changes in corrected calcium levels divided by saline solution administration.

Supplementary Figure 3. Changes in corrected calcium levels divided by bisphosphonate administration.

Supplementary Figure 4. Changes in corrected calcium levels for patients administered with a combination treatment of saline solution and bisphosphonates.
